# Bovine Genomics From Academia to Industry

**DOI:** 10.1002/cfg.467

**Published:** 2005-04

**Authors:** David Henderson, Milt Thomas, Yang Da

**Affiliations:** 1 231 Shantz Building Department of Animal Sciences The University of Arizona Tucson AZ 85721 USA; 2 Department of Animal and Range Sciences New Mexico State University Las Cruces NM 88003 USA; 3 Department of Animals Sciences University of Minnesota St. Paul MN 55108-6118 USA


**Comparative and Functional Genomics**
*Comp Funct Genom* 2005; **6**: 174–180.
Published online in Wiley InterScience (www.interscience.wiley.com). **DOI:** 10.1002/cfg.467

**Conference Paper**

### Conference review: bovine genomics from academia to industry

### Cattle and Sheep Workshop, Plant & Animal Genomes (PAG) XIII Conference, San Diego, CA, USA

David Henderson[1]*, Milt Thomas[2] and Yang Da[3]

[1] *Department of Animal Sciences, The University of Arizona, Tucson, AZ 85721, USA*
[2] *Department of Animal and Range Sciences, New Mexico State University, Las Cruces, NM 88003, USA*
[3] *Department of Animals Sciences, University of Minnesota, St. Paul, MN 55108-6118, USA*

**Correspondence to: David Henderson, 231 Shantz Building, Department of Animal Sciences, The University of Arizona, Tucson, AZ 85721, USA. E-mail: DNADave@U.Arizona.Edu*

Received: 31 January 2005
Accepted: 2 February 2005

### Introduction

Two multistate United States Agricultural Experiment Station projects, North Central (NC)-1010 and National Research Support Project (NRSP)-8, collaborated on a joint presentation at the PAGXIII conference in San Diego, CA. The joint session consisted of presentations on current and future technologies for ruminant research. Topics ranged from genome discovery to tool applications. The current state of the art in bovine and ovine research is near that seen in the model organisms, although research methods may be a few steps back of many near future technologies that researchers of model organisms are currently developing (see the section on Trey Ideker for an example). Current problems that need immediate attention are those of implementation of research findings and education of producers as to how they might implement the results of quantitative trait loci (QTL) scans and candidate gene searches. The outcomes of the committee indicate that bioinformatics and computational biology approaches in both tool development and tool usage will be major areas of emphasis for the next few years. The following review will highlight information that was presented in the areas of genetic markers, gene mapping and sequencing, bioinformatics, functional genomics, as well as industry perspectives. Summaries of the NC-1010 and NRSP-8 committees will also be presented.

### Bovine sequencing efforts

**Jim Womack** from Texas A&M University gave a detailed presentation entitled 'Highlights of the bovine sequencing project'. The sequence of the human genome was published in 2001 [23] and this publication necessitated sequencing of a bovine genome for both agricultural purposes and for use of the bovine as a model organism to study human health. The following organizations contributed to the project:

- National Human Genome Research Institute ($25 million).

Copyright © 2005 John Wiley & Sons, Ltd.

- USDA-National Research Inititiative ($10 million).
- State of Texas ($8 million).
- Genome Canada ($5 million).
- Kleberg Association ($2 million).
- USDA-ARS ($1 million).
- CSIRO Australia ($1 million).
- New Zealand ($1 million).
- United States Beef Council ($0.4 million).
- Texas Beef Council and South Dakota Beef Council (<$0.2 million).

Jim Womack reviewed the discussions towards sequencing a bovine genome, which were started as early as 1993 by the cattle and sheep discussion group known as NRSP-8. This project, which is known as the International Bovine Genome Sequencing Project, was initiated in 2003 and is directed by a steering committee. Much of the initial sequencing effort is occurring at the Human Genome Sequencing Center at Baylor College of Medicine in Houston, TX. Richard Gibbs and George Weinstock are directing these efforts and a $\sim 3\times$ working draft was published in October of 2004 and is available at GenBank. The animal resources from this project were derived from the historic linebreeding Hereford project at the Fort Keogh US Department of Agriculture–Agriculture Research Service (USDA-ARS) station in Miles City, Montana [12,13,19]. A bacterial artificial chromosome (BAC) DNA library was originally created from tissues of the bull L1 Domino 99 375, and the whole genome shotgun sequences were derived from DNA extracted from the white blood cells of his daughter, L1 Dominette 01 449. Dominette was selected since her sire was used to create the BAC library and because of the concept that sequence assembly could be made easier working with sequences from animals with similarities in their genetic background. Other contributions to this project now include sequencing within other breeds such as Holstein, Angus, Brahman, Jersey, Limousine and Norway Red. Some of these other efforts include SNP mapping, as well as $\sim$10 000 full-length mRNA sequences to be provided by Genome Canada. A 7–8$\times$ coverage map is expected by late summer of 2005.

### Bioinformatics

**Trey Ideker** of the University of California, San Diego, gave a presentation entitled 'Modeling cells with molecular interaction networks'. Molecular interaction networks are experimentally derived connections of metabolites and proteins within pathways. While these molecules may not directly interact, they likely are related through the enzymes that process them. Dr Ideker presented evidence that overlays, the alignment of pathways according to protein sequence or other data where similarity scores are available, may be of great use. Specifically, overlays of known networks from well-studied species may elucidate the function or role of unknown metabolites and proteins in less well understood organisms. The implications of this research involve an increase in the speed of which function can be ascribed to unknown compounds in bovine, using information gathered in human, mouse and rat.

**Christian Looft** of the University of Kiel, Germany, presented material on the 'Structural and functional analysis of antimicrobial peptides in the bovine mammary gland'. Dr Looft was interested in the expression of $\beta$-defensin genes within the bovine mammary gland. Following a genomic characterization of defensin genes, seven novel $\beta$-defensin genes and four pseudo-genes were identified and mapped in two BAC contigs that spanned approximately 700 kb. Another antimicrobial peptide, psoriasin, was investigated based upon the functionality of the human homologue for anti-*E. coli* activity. The peptide was isolated from bovine dander and characterized by MS. In an ironic twist of fate, an *E. coli* was used to replicate a recombinant version of the peptide. The group was able to demonstrate the anti- *E. coli* activity of this peptide when presented outside of the cell membrane, while showing that the peptide is not able to kill *E. coli* cells while still inside the cell membrane.

**Christine Elsik** of Texas A&M University (TAMU) spoke on the current state of the bovine long oligo array developed among a consortium of researchers at TAMU, University of Missouri, Iowa State University, and The University of Minnesota. Sequences mined from GenBank are currently undergoing a screening process to remove duplicated sequences, vector sequences and other artifacts of the cloning process. It is expected that a first draft of the sequences to be spotted on the array will be available by Summer 2005.

Copyright © 2005 John Wiley & Sons, Ltd.

### Functional genomics

**Mark Jutila** (Montana State University) presented results of functional genomics analysis of bovine gammadelta and $\alpha\beta$ T cells from USDA-Initiative for Future Agriculture and Food Systems (IFAFS)- and National Institutes of Health (NIH)-funded projects in collaboration with Mitchell Abrahamsen (University of Minnesota) [7,8,14]. Changes in bovine $\gamma\delta$ and $\alpha\beta$ T cells in the draining lymphatics of the gut mucosa following *S. typhimurium* infection were determined by fluorescent activated cell sorting (FACS) and microarray analyses. Cannulae were inserted into the mesenteric lymphatic ducts of calves for collection of cells draining from the intestine and lymphatic fluid collected at intervals pre- and post-infection. In the blood of calves with a fever and diarrhoea, neutrophils, monocytes and B cells increased slightly during the course of the 72 h infection, whereas in the lymphatic fluid, $\gamma\delta$ T cells steadily increased in frequency and all other cell types decreased or remained constant. During two such procedures, the $\gamma\delta$ and $\alpha\beta$ T cells were purified in sufficient numbers for RNA isolation and genomic analysis. Bovine cDNA (Pyxis Cattle 7600) and Affymetrix bovine oligonucleotide arrays were utilized to elucidate the changes in gene expression at 0, 6 and 24 h after *S. typhimurium* infection in both T cell subsets. The magnitude of the response in both T cell types was comparable, but the genes that changed were different. The Affymetrix arrays allowed for comparison over the time course within one subset, as well as comparison between cell types, and revealed more than 1000 genes differentially expressed between $\gamma\delta$ and $\alpha\beta$ T cells at 0 and 6 h post-infection. Affymetrix arrays also demonstrated the expression of known immune related genes suggesting distinct functions of the T cell subsets during early *S. typhimurium* infection.

### Animal traceability, gene and QTL mapping

**Michael Heaton** of the USDA-ARS Meat Animal Research Center (MARC) presented material on 'bovine single nucleotide polymorphisms (SNP)s: animal identification, traceback and pedigree verification' in the NC1010 symposium. Parent verification and the ability to trace animal products using DNA polymorphism procedures are now being utilized by the livestock industries. Microsatellites were the original technology for these procedures; however, usable SNPs have now been published [6], with another publication in press in the *Journal of the American Veterinary Medical Association* soon to assist these findings. These procedures proved valuable when bovine spongiform encephalopathy (BSE) was detected in the USA in December 2003. A team of scientists representing the USDA-ARS and two commercial genotyping labs developed assays so that in a time span of 48–72 h, field veterinarians and staff of USDA-Animal Plant Health Inspection Service (APHIS) could trace meat products from slaughter facility to farm.

**Stephen White** from USDA-ARS MARC presented a talk on 'New SNP marker in CAPN-1 associated with tenderness in cattle of indicine, taurine and admixed descent'. There is tremendous interest in the trait of tenderness in the basic scientific community and in the commercial genotyping industry. A challenge in the application of some of the initially discovered polymorphisms related to tenderness was that the markers inferred variability in prediction of tenderness traits and had diversity among genotypes in *Bos taurus* cattle, but not in *Bos indicus* cattle [3,17,18]. These studies revealed that chromosome 29 contained an important gene defined as $\mu$-calpain (CAPN-1), which has a large role in post-mortem tenderization. There are a series of SNPs within or closely linked to this gene. Two of these SNPs appear to be informative in *Bos indicus* and *Bos taurus* cattle (316 and 4753), but a SNP at 530 only appears to be informative in *Bos taurus* cattle. Moreover, it appears that these markers maybe more useful in predictions using analytical procedures involving haplotypes.

**Stephen Moore** from the University of Alberta at Edmonton delivered a talk on 'Candidate genes of feed efficiency'. The trait of residual feed intake was introduced [9] and efforts to find genetic tools to select for this trait were discussed. Data on other measures of animal efficiency, which involved evaluating oxygen consumption and methane production using a respiratory calorimetry hood, were also presented. The rationale for these measures comes from the concept that measures of gas consumption or production could be used to estimate metabolic rate/efficiency in cattle. Efforts to

Copyright © 2005 John Wiley & Sons, Ltd. 

use these measures to identify candidate genes were also presented, particularly their associations with hormones such as leptin [16].

**Jeremy Taylor** (University of Missouri) reported results in fine quantitative trait loci (QTL) mapping in dairy cattle (in collaboration with scientists at USDA-ARS and the University of Arizona), as well as results in positional cloning in beef cattle. To fine-map QTL affecting milk production traits in dairy cattle on BTA6 [1], 3317 bulls comprising 45 half-sib families were genotyped for 38 markers. The data were analysed using least squares regression (QTL Express), linkage disequilibrium (LDVCM) and full pedigree MCMC (LOKI) methods. A total of 19 sires were segregating for at least one QTL under a half-sib model at chromosome-wide $p < 0.05$. LD results across families indicated the presence of up to seven QTLs (three within a 6 cM region), whereas LOKI revealed only three QTLs. Positional/functional candidate genes have been identified for five of the QTLs. Osteopontin (OPN or SPP1) is a strong candidate for the QTL near marker BM143. The entire OPN gene and 5 kb upstream was sequenced from four segregating sires and four non-segregating sires (12.3 kb/animal). A total of 15 SNPs were identified but only one SNP resulted in sire genotypes that were concordant with the segregation status of all eight sires. For position cloning in beef cattle, the strategy outlined in Taylor and Schnabel [20] was applied and built upon the previous projects involving *Bos taurus* × *Bos indicus* crosses [10] and study of *Bos taurus* autosomal (BTA) chromosomes 2 and 14 [5,11,15]. A DNA repository from the semen of 1660 registered bulls representing 14 generations of the American Angus Association was assembled. Expected progeny differences (EPDs) and reliabilities for 20 traits are available for this population. In addition, 5300 DNA samples were collected on commercial Angus steers (36 half-sib families have at least 30 progeny) with growth and slaughter phenotypes. A total of 113 637 genotypes for 56 microsatellite loci and SNPs for thyroglobulin in exon 5 (TG5) and diacyl glycerol acyltransferase in exon 1 (DGAT1) on BTA2 and BTA14 in 1361 Angus sires and 559 steers were scored. The genoprob program of Thallman *et al.* [21,22] assisted in the pedigree analyses. The results suggested that TG5 had no effect on sire marbling EPDs or steer marbling phenotypes. Three previously published marbling QTLs, one birth weight QTL and one carcass weight QTL are segregating within Angus and map to identical locations to the published reports.

### Industry perspective

**John Pollack** from Cornell University spoke on NBCEC efforts in marker validation. The National Beef Cattle Evaluation Consortium (NBCEC) is a consortium of four universities involved in genetic evaluation of beef cattle breeds. Colorado State, Cornell, Iowa State and Georgia are the participating universities. The consortium is supported by a federally funded special grant and part of the funds from that grant are used to support educational programs (**http://www.ansci.cornell.edu/nbcec/index.html**). Because of the role of the consortium in national cattle evaluation and recent introduction of marker-assisted EPDs (**http://www.simmental.org/ASA_Today/Cow%20Awards%20Folders/MAEPDs.html**), NBCEC has formed a QTL team to develop procedures for validating commercialized genetic markers. Members of the QTL team are R. L. Quass of Cornell University, R. M. Thallman of USDA-ARS-MARC, and R. L. Fernando of Iowa State University. In general, the consortium will conduct blind testing of markers in resource populations. The current test populations involve DNA and data from the National Cattlemen's Beef Association (NCBA) Carcass Merit Project, outreach projects of NBCEC involving the Bell and King ranches, and cycles 7 and 8 of the USDA-ARS Germplasm Evaluation Program [4]. The consortium is seeking samples and data from other research and resource populations, as they would like to develop a repository with ample representation of British, Continental and *Bos indicus*-influenced breeds. Dr Pollock stated that 'there are three challenges currently facing the beef cattle industry with the use of genetic marker information for genetic improvement programs: (a) DNA marker information is not being adequately collected by breed associations; (b) there are no policing mechanisms for use and interpretation of DNA marker information; and (c) assessment of markers is not currently part of the process of evaluation of DNA markers.

Copyright © 2005 John Wiley & Sons, Ltd.

**Michael Cowan** (Accelerated Genetics) discussed his company's practice and results of applying marker-assisted selection in dairy cattle. His company started applying marker techniques to genetic selection in 1988 by screening impact sire families, using polymorphisms in known genes, with the goal of improving the accuracy in selecting young bulls prior to entering progeny-testing programs [2]. Restriction fragment length polymorphisms (RFLP) in the prolactin/placental lactogen gene family and gene markers involved in immunity and lactation (DRB and CYP21) haplotypes were screened for three sire families for their effects on the predicted transmitting ability (PTA) of milk, fat and protein yields, as well as composite yields measured by dollars, and found significant marker effects. Full sib comparisons showed significant improvement in the realized gains in the above traits. The company also used genetic markers to monitor semen processing, parentage identification and inheritance of new genetic defects. To improve the efficiency of marker-assisted selection in the dairy industry, more work is needed in the following areas: characterization of additional bovine clones and regulatory sequences, development of methods to rapidly screen genomes of multiple individuals, better understanding of gene function and interactions, and more emphasis on traits with low heritabilities.

**Marjorie Faust** from ABS Global Inc., spoke on 'How do we move from the laboratory to the bull barn?' QTL mapping efforts have delivered many promising candidate genes for selection within the Holstein breed, or other bovine breeds in general. There is little consistency in how these genes are utilized, or characterized within commercial populations. The producer often lacks the technical expertise to master the nuances of effect and efficacy of each candidate gene commonly understood by the scientific community. Better efforts to appropriately educate the producer and to evaluate potential gene targets for selection in relevant populations are necessary for marker-assisted selection to succeed as a commonly used commercial tool. It also appears that the cost of implementing marker-assisted selection is the role of the semen stud rather than the organization wanting to benefit from the new selection tool. The industry currently would like to pay less per unit of semen, which results in limited dollars for the cost of genotyping.

### Summary of NSRP-8 station report: cattle, sheep and goats

The objectives of this project NSRP-8 are:

1. Enhance and integrate genetic and physical maps of agriculturally important animals for cross-species comparisons and sequence annotation.
2. Facilitate integration of genomic, transcriptional, proteomic and metabolomic approaches toward better understanding of biological mechanisms underlying economically important traits.
3. Facilitate and implement bioinformatics tools to extract, analyse, store and disseminate information.

Scientists from the University of Arkansas, the University of California at Davis, the University of Illinois, Iowa State University, Louisiana State University, Purdue University, New Mexico State University, Oklahoma State University, Texas A&M University, Utah State University, the University of Wisconsin–Madison and a few other universities gathered to present and discuss information regarding these objectives. The scope of the information ranged from mapping projects and defining QTL to presentation of the effects of polymorphisms in candidate genes in cattle and sheep challenged with different production systems and environments. Genetic influences of lactation and behavioural traits such as disposition were also discussed, as were the influences of genetic anomalies, such as callipyge. Milt Thomas of New Mexico State University served as the session coordinator and was assisted by secretary Udaya Desilva of Oklahoma State University, who will serve as the session coordinator in 2006. Clare Gill of Texas A&M University was elected to serve as the secretary in 2006. Bioinformatic information was also presented and the committee voted to include discussion of goats into the symposium.

### Summary of NC-1010 station report

The objectives of this multi-state project are:

1. Determine the location, structure, function and expression of genes affecting health, reproduction, production and product quality in cattle.

Copyright © 2005 John Wiley & Sons, Ltd. 

2. Interpret and apply genomics and proteomics information by developing statistical/bioinformatics methods and utilizing molecular tools in cattle.
3. Develop and deliver educational materials about bovine genomics research to consumers and stakeholders.

Scientists from the University of Arizona, the University of California at Davis, Michigan State University, Texas A&M University, the University of Vermont, the University of Illinois (Urbana–Champagne), Ohio State University, the University of Minnesota and the University of Wisconsin were represented. Alison van Eenennaam of UC–Davis served as chair of the session, assisted by secretary David Henderson of the University of Arizona. Primary discussion centred on the development of educational material on biotechnology aimed at producers. The chosen medium was the World-Wide-Web and it is to be hosted at UC–Davis. David Henderson will serve as chair next year and Clare Gill of Texas A&M University will serve as secretary next year.

This project would like to:

1. Continue development of educational material on biotechnology.
2. Develop material for a booth at the 2006 American Society of Animal Scientists/American Dairy Science Association meeting and conduct a graduate student poster session at the next meeting of NC-1010.

### Conclusions

Biotechnology research of the bovine and ovine is lagging behind that of the basic model organisms only in the understanding of the organism; however, now that sequence is becoming available, knowledge of these species will accelerate. QTLs for many production and quality traits are either mapped or are being mapped and candidate genes for selection are under investigation. Future work in the model organisms is likely to produce new tools that will help elucidate the underlying biology behind such complicated biological processes as feed conversion and efficiency to marbling and meat quality. Defining these technical developments is needed, to educate producers on the use and importance of genomic technologies in modern agricultural practice.

### Acknowledgements

The authors would like to acknowledge the Agricultural Experimental Stations of the University of Arizona, New Mexico State University and the University of Minnesota, and the Agricultural Experiment Station efforts in the committees of NC1010 (Interpreting Cattle Genomic Data: Biology, Applications and Outreach) and NRSP-8 (National Animal Genome Research Program — National Research Support Project 8) focused on cattle, sheep and goats.

### References

1. Ashwell MS, Heyen DW, Sonstegard TS, *et al.* 2004. Detection of quantitative trait loci affecting milk production, health, and reproductive traits in Holstein cattle. *J Dairy Sci* **87**: 468–475.
2. Cowan CM, Dentine MR, Coyle T. 1992. Chromosome substitution effects associated with $\kappa$-Casein and ß-lactoglobulin in Holstein cattle. *J Dairy Sci* **75**: 1097–1104.
3. Casas E, White SN, Riley DG, *et al.* 2005. Assessment of single nucleotide polymorphisms in genes residing on chromosome 14 and 29 for association with carcass composition traits in *Bos indicus* cattle. *J Anim Sci* **83**: 13–19.
4. Cundiff LV, Szabo F, Gregory KE, *et al.* 1999. Breed comparison in the germplasm evaluation program at MARC. USDA-MARC GPE Prog. Rep. No. 22. USDA.
5. Grosz MD, MacNeil MD. 2001. Putative quantitative trait locus affecting birth weight on bovine chromosome 2. *J Anim Sci* **79**: 68–72.
6. Heaton MP, Harhay GP, Bennett GL, *et al.* 2002. Selection and use of SNP markers for animal identification and paternity analysis in US beef cattle. *Mamm Genome* **13**: 272–281.
7. Hedges JF, Cockrell D, Jackiw L, *et al.* 2003. Differential mRNA expression in circulating $\gamma\delta$ T lymphocyte subsets defines unique tissue-specific functions. *J Leuk Biol* **73**: 306–314.
8. Hedges JF, Graf J, Jutila MA. 2003. Transcriptional profiling of $\gamma\delta$ T cells. *J Immunol* **171**: 4959–4964.
9. Kennedy BW, van der Werf HJ, Meuwissen THE. 1993. Genetic and statistical properties of residual feed intake. *J Anim Sci* **71**: 3239–3250.
10. Kim JJ, Farnir F, Savell J, Taylor JF. 2003. Detection of quantitative trait loci for growth and beef carcass fatness traits in a cross between *Bos taurus* (Angus) and *Bos indicus* (Brahman) cattle. *J Anim Sci* **81**: 1933–1942.
11. Li C, Basarab J, Snelling WM, *et al.* 2004. Identification and fine mapping of quantitative trait loci for backfat on bovine chromosomes 2, 5, 6, 19, 21 and 23 in a commercial line of *Bos taurus*. *J Anim Sci* **82**: 967–972.
12. MacNeil MD, Dearborn DD, Cundiff LV, Dinkel CA, Gregory KE. 1989. Effects of inbreeding and heterosis in Hereford females on fertility, calf survival and preweaning growth. *J Anim Sci* **67**: 895–901.

Copyright © 2005 John Wiley & Sons, Ltd.

13. MacNeil MD, Urick JJ, Newman S, Knapp BW. 1992. Selection for postweaning growth in inbred Hereford cattle: the Fort Keogh, Montana line 1 example. *J Anim Sci* **70**: 723–733.
14. Meissner N, Radke J, Hedges JF, *et al*. 2003. Serial analysis of gene expression in circulating $\gamma\delta$ T cell subsets defines distinct immunoregulatory phenotypes and unexpected gene expression profiles. *J Immunol* **170**: 356–364.
15. Moore SS, Li C, Basarab J, *et al*. 2003. Fine mapping of quantitative trait loci and assessment of positional candidate genes for backfat on bovine chromosome 14 in a commercial line of *Bos taurus*. *J Anim Sci* **81**: 1919–1925.
16. Nkrumah JD, Li C, Yu J, *et al*. 2005. Polymorphisms in the bovine leptin promoter associated with serum leptin concentration, growth, feeding behavior, and measures of carcass merit. *J Anim Sci* **83**: 20–28.
17. Page BT, Casas E, Heaton MP, *et al*. 2002. Evaluation of single-nucleotide polymorphisms in CAPN1 for association with meat tenderness in cattle. *J Anim Sci* **80**: 3077–3085.
18. Page BT, Casas E, Quaas RL, *et al*. 2004. Association of markers in the bovine CAPN1 gene with meat tenderness in large crossbred populations that sample influential industry sires. *J Anim Sci* **82**: 3474–3481.
19. Pariacote F, Van Vleck LD, MacNeil MD. 1998. Effects of inbreeding and heterozygosity on preweaning traits in a closed population of Herefords under selection. *J Anim Sci* **76**: 1303–1310.
20. Taylor JF, Schnabel RD. 2004. Prospects for positional cloning of QTL in general pedigrees using the bovine whole genome sequence. Satellite Symposium to the 29th International Conference on Animal Genetics, Japanese Society of Animal Breeding and Genetics, Tokyo, Japan, 16–17 September.
21. Thallman RM, Bennett GL, Keele JW, Kappes SM. 2001. Efficient computation of genotype probabilities for loci with many alleles: I. Allelic peeling. *J Anim Sci* **79**: 26–33.
22. Thallman RM, Bennett GL, Keele JW, Kappes SM. 2001. Efficient computation of genotype probabilities for loci with many alleles: II. Iterative method for large, complex pedigrees. *J Anim Sci* **79**: 34–44.
23. Venter JC, Adams MD, Myers EW, *et al*. 2001. The sequence of the human genome. *Science* **291**: 1304–1351.

Copyright © 2005 John Wiley & Sons, Ltd. 

## References

[PDF_00524] Ashwell M. S., Heyen D. W., Sonstegard T. S., Van Tassell C. P., Da Y., VanRaden P. M., Ron M., Weller J. I., Lewin H. A. (2004). Detection of quantitative trait loci affecting milk production, health, and reproductive traits in Holstein cattle.. J Dairy Sci.

[PDF_00531] Casas E., White S. N., Riley D. G., Smith T. P. L., Brenneman R. A., Olson T. A., Johnson D. D., Coleman S. W., Bennett G. L., Chase C. C. (2005). Assessment of single nucleotide polymorphisms in genes residing on chromosomes 14 and 29 for association with carcass composition traits in Bos indicus cattle.. J Anim Sci.

[PDF_00528] Cowan C. M., Dentine M. R., Coyle T. (1992). Chromosome substitution effects associated with kappa-casein and beta-lactoglobulin in Holstein cattle.. J Dairy Sci.

[PDF_00538] Grosz M. D., MacNeil M. D. (2001). Putative quantitative trait locus affecting birth weight on bovine chromosome 2.. J Anim Sci.

[PDF_00541] Heaton Michael P., Harhay Gregory P., Bennett Gary L., Stone Roger T., Grosse W. Michael, Casas Eduardo, Keele John W., Smith Timothy P. L., Chitko-McKown Carol G., Laegreid William W. (2002). Selection and use of SNP markers for animal identification and paternity analysis in U.S. beef cattle.. Mamm Genome.

[PDF_00544] Hedges Jodi F., Cockrell Diane, Jackiw Larissa, Meissner Nicole, Jutila Mark A. (2003). Differential mRNA expression in circulating gammadelta T lymphocyte subsets defines unique tissue-specific functions.. J Leukoc Biol.

[PDF_00548] Hedges Jodi F., Graff Jill C., Jutila Mark A. (2003). Transcriptional profiling of gamma delta T cells.. J Immunol.

[PDF_00550] Kennedy B. W., van der Werf J. H., Meuwissen T. H. (1993). Genetic and statistical properties of residual feed intake.. J Anim Sci.

[PDF_00553] Kim J. J., Farnir F., Savell J., Taylor J. F. (2003). Detection of quantitative trait loci for growth and beef carcass fatness traits in a cross between Bos taurus (Angus) and Bos indicus (Brahman) cattle.. J Anim Sci.

[PDF_00557] Li C., Basarab J., Snelling W. M., Benkel B., Kneeland J., Murdoch B., Hansen C., Moore S. S. (2004). Identification and fine mapping of quantitative trait loci for backfat on bovine chromosomes 2, 5, 6, 19, 21, and 23 in a commercial line of Bos taurus.. J Anim Sci.

[PDF_00561] MacNeil M. D., Dearborn D. D., Cundiff L. V., Dinkel C. A., Gregory K. E. (1989). Effects of inbreeding and heterosis in Hereford females on fertility, calf survival and preweaning growth.. J Anim Sci.

[PDF_00567] MacNeil M. D., Urick J. J., Newman S., Knapp B. W. (1992). Selection for postweaning growth in inbred Hereford cattle: the Fort Keogh, Montana line 1 example.. J Anim Sci.

[PDF_00571] Meissner Nicole, Radke Jay, Hedges Jodi F., White Michael, Behnke Michael, Bertolino Shannon, Abrahamsen Mitchell, Jutila Mark A. (2003). Serial analysis of gene expression in circulating gamma delta T cell subsets defines distinct immunoregulatory phenotypes and unexpected gene expression profiles.. J Immunol.

[PDF_00575] Moore S. S., Li C., Basarab J., Snelling W. M., Kneeland J., Murdoch B., Hansen C., Benkel B. (2003). Fine mapping of quantitative trait loci and assessment of positional candidate genes for backfat on bovine chromosome 14 in a commercial line of Bos taurus.. J Anim Sci.

[PDF_00579] Nkrumah J. D., Li C., Yu J., Hansen C., Keisler D. H., Moore S. S. (2005). Polymorphisms in the bovine leptin promoter associated with serum leptin concentration, growth, feed intake, feeding behavior, and measures of carcass merit.. J Anim Sci.

[PDF_00583] Page B. T., Casas E., Heaton M. P., Cullen N. G., Hyndman D. L., Morris C. A., Crawford A. M., Wheeler T. L., Koohmaraie M., Keele J. W. (2002). Evaluation of single-nucleotide polymorphisms in CAPN1 for association with meat tenderness in cattle.. J Anim Sci.

[PDF_00586] Page B. T., Casas E., Quaas R. L., Thallman R. M., Wheeler T. L., Shackelford S. D., Koohmaraie M., White S. N., Bennett G. L., Keele J. W. (2004). Association of markers in the bovine CAPN1 gene with meat tenderness in large crossbred populations that sample influential industry sires.. J Anim Sci.

[PDF_00590] Pariacote F., Van Vleck L. D., MacNeil M. D. (1998). Effects of inbreeding and heterozygosity on preweaning traits in a closed population of Herefords under selection.. J Anim Sci.

[PDF_00600] Thallman R. M., Bennett G. L., Keele J. W., Kappes S. M. (2001). Efficient computation of genotype probabilities for loci with many alleles: I. Allelic peeling.. J Anim Sci.

[PDF_00603] Thallman R. M., Bennett G. L., Keele J. W., Kappes S. M. (2001). Efficient computation of genotype probabilities for loci with many alleles: II. Iterative method for large, complex pedigrees.. J Anim Sci.

[PDF_00607] Venter J. C., Adams M. D., Myers E. W., Li P. W., Mural R. J., Sutton G. G., Smith H. O., Yandell M., Evans C. A., Holt R. A. (2001). The sequence of the human genome.. Science.

